# Satisfaction and quality of life in cochlear implant users with long sensory deprivation

**DOI:** 10.1590/2317-1782/20232021021en

**Published:** 2023-07-17

**Authors:** Alleluia Lima Losno Ledesma, Kétlim dos Santos Evangelista, Driely Maria Leandro de Alexandria, Jéssica da Silva Sales, Fernanda Ferreira Caldas, Fayez Bahmad

**Affiliations:** 1 Programa de Pós-graduação em Ciências da Saúde, Universidade de Brasília – UnB - Brasília (DF), Brasil.; 2 Faculdade de Fonoaudiologia, Centro Universitário Planalto do Distrito Federal – UNIPLAN - Brasília (DF), Brasil.

**Keywords:** Cochlear Implants, Patient Satisfaction, Quality of Life, Hearing Loss, Hearing Loss, Sensorineural, Implante Coclear, Satisfação do Paciente, Qualidade de Vida, Perda Auditiva, Perda Auditiva Neurossensorial

## Abstract

**Purpose:**

The aim of the study was to verify the level of satisfaction of CI users with long periods of hearing deprivation, highlighting the positive and negative aspects of the use of the device and their quality of life.

**Methods:**

This is a analytical research, of the type observational cross-sectional study. The study was performed with 24 patients from a private Institute of Otorhinolaryngology. Three surveys were applied: Satisfaction with Amplification in Daily Life (SADL), International Outcome Inventory - Cochlear Implant (IOI - CI) to assess cochlear implant satisfaction and WHOQOL- bref to assess the quality of life. The results in the pre and post lingual groups were compared.

**Results:**

The highest degree of satisfaction was reported with regard to personal image, positive effects, and how the users feel about their CI. The lowest degree of satisfaction was reported regarding the cost-benefit of the CI and the competitive noise. In the WHOQOL-bref assessment, the highest scores were found in physical, psychological and social relations domains. When comparing the results of the surveys, the pre and post-lingual groups showed no difference in relation to the achieved scores.

**Conclusion:**

The participants had a high level of satisfaction with the use of cochlear implants. The longer the sensory deprivation time, the greater the degree of satisfaction with the device. The use of the CI electronic device reflects on the individual's quality of life.

## RESUMO

**Objetivo:**

Verificar o nível de satisfação de usuários de IC com longos períodos de privação auditiva, destacando os aspectos positivos e negativos do uso do dispositivo e avaliar a qualidade de vida desses indivíduos.

**Método:**

Trata-se de uma pesquisa analítica, do tipo estudo observacional transversal. O estudo foi realizado com 24 pacientes de um instituto privado de Otorrinolaringologia. Foram aplicados três questionários: Satisfaction with Amplification in Daily Life (SADL), International Outcome Inventory- Implante Coclear (IOI - IC) para avaliar a satisfação com o implante coclear e WHOQOL-bref para avaliar a qualidade de vida. Os resultados nos grupos pré e pós-lingual foram comparados.

**Resultados:**

O maior grau de satisfação foi relatado em relação à imagem pessoal, efeitos positivos e como os usuários se sentem em relação ao seu IC. O menor grau de satisfação ocorreu em relação ao custo-benefício do IC e ao ruído competitivo. Na avaliação do WHOQOL-bref, os maiores escores foram encontrados nos domínios físico, psicológico e relações sociais. Os grupos pré e pós-lingual não apresentaram diferença em relação aos escores alcançados.

**Conclusão:**

Os participantes apresentaram alto nível de satisfação com o uso do implante coclear. Quanto maior o tempo de privação sensorial maior o grau de satisfação com o dispositivo. O uso do dispositivo eletrônico de IC reflete melhora na qualidade de vida do indivíduo.

## INTRODUCTION

Hearing loss (HL) can negatively impact the quality of life of adults^(1)^. It may be associated with social isolation, impairment of speech, psychological and professional development, as well as feelings of disability and inferiority^(2)^. These factors can lead to emotional problems such as low self-esteem, loneliness, depression, and irritability^(2)^. Study with adults with bilateral hearing loss (HL) related the difficulty in maintaining interpersonal relationships due to hearing impairment with low quality of life^(3)^.

Part of individuals with HL who have partial functioning of the ear hair cells may acquire relevant gain with the use of hearing aids. While those who do not benefit from this may be indicated for cochlear implant (CI) surgery as an alternative form of treatment^(4)^.

The CI is an electronic device whose function is to partially replace the activities of Corti's organ, by means of electrical impulses that directly stimulate the remaining neural fibers of the cochlea. It consists of two parts. The internal part consists of a bundle of electrodes that are inserted into the cochlea and a stimulating receptor positioned surgically in the region of the temporal bone. The external part consists of a signal processor, a microphone and a transmitting antenna located in a retroauricular position that is responsible for capturing the sound that will be transmitted to the speech processor^(5)^.

Several medical entities throughout the world have published recommendations for selecting candidates for cochlear implant^(6-8)^. There is general consensus in these publications about the candidacy of CI in adults: having severe-profound hearing loss, little gain with the use of hearing aids and evaluation by a multidisciplinary team.

In Brazil, the availability of CI technology is a reality in both the private and public health sectors, and the number of CI users has been increasing over the years^(9)^.

CI is considered a well-established treatment for severe-profound sensorineural hearing loss. Despite this, there is little study on the satisfaction level in users of this technology in individuals who had had long periods of hearing deprivation. In addition to satisfaction, it is important to consider how improving hearing perception impacts the quality of life of users^(10)^.

It is reported be significantly improves the quality of life after cochlear implantation; however, few studies relate these results in users with longer time of hearing loss before cochlear implantation^(11)^. It is suggested that quality of life and speech recognition in adults with prelingual deafness improved significantly after cochlear implantation^(12,13)^. Contrary to expectations, these later studies revealed more benefit in the quality of life in users of cochlear implant with longer pre-implant hearing loss, suggesting that this result can be explained by the implication of reentering the world of hearing. The authors added, further, that the data currently available cannot fully explain this result^(14)^.

Thus, the aim of this study is to verify the level of satisfaction of CI users who had had long periods of hearing deprivation, highlighting the positive and negative aspects of the use of the electronic device and their overall quality of life.

## METHODS

### Study design

This is an analytical research, an observational cross-sectional study.

### Ethics committee approval

This research was submitted to the Ethics Committee, approved with CAAE: 70324117.0.0000.5650. The sample consisted of patients from a Cochlear Implant Center. All participants signed the written informed consent.

### Inclusion and exclusion criteria

Inclusion criteria were: severe/profound, pre or postlingual sensorineural hearing loss with long sensory deprivation (ten years or more)^(15)^, being over 18 years old, having had implant activation within a minimum period of 6 months, literate and capable of answering to the surveys.

Exclusion criteria were: Individuals with cognitive impairment or some other associated disability or those with shorter duration of hearing.

### Selection criteria used for cochlear implant indication recommendation

The selected patients were in accordance with the guidelines “Cochlear Implant Indicator Criteria” elaborated by the consensus of the Brazilian Association of Otorhinolaryngology and Facial Cervical Surgery, Brazilian Society of Otology, Brazilian Society of Speech Therapy, Brazilian Academy of Audiology and Brazilian Society of Pediatrics. It is recommended cochlear implants for patients with bilateral profound sensorineural hearing loss with proven limited benefits with the hearing aids bilaterally.

That is:

- Severe or profound bilateral sensorineural hearing loss;
- Result equal to or less than 50% of sentence recognition in open set with the use of hearing aids in both ears;
- Presence of language code established and adequately rehabilitated by the oral method;
- The adequate motivation of the patient for the use of cochlear implant and for the speech rehabilitation process

The implanted patients who met the study selection criteria were invited to participate in the research at the same time as their therapy and/or medical return visits. After the documented acceptance, they answered a case study, two inventories about satisfaction with the use of the CI, and an assessment about their quality of life.

### Selection of participants

The sample of the present study was of convenience, being selected from the service's database the patients who met the research selection criteria and still had a connection with the service. Participants were invited to participate in the research, which was carried out before or after a routine visit to the institute.

### Surveys: IOI – CI, SADL, WHOQOL-bref

The surveys were: International Outcome Inventory - Cochlear Implant (IOI - CI) and Satisfaction with Amplification in Daily Life (SADL), which were originally developed for users of hearing aids, but were adapted for CI^2^ users. And the WHOQOL-bref assessment that evaluates the quality of life in predetermined aspects^(16)^.

The IOI-CI inventory has seven questions to which the answer is a 5-point likert scale, where low values indicate worse results. It consists of two factors: factor 1 (4 questions) indicates the user's relationship with the use of their device (eg: usage time, CI efficiency to hear better, is CI worth using) and factor 2 (3 questions) reflects the user's interaction with the social environment (eg, relationships with other people, in activities and the enjoiment of life has changed). As a global result, the sum of the scores of factors 1 and 2 is performed. The arithmetic mean of the scores obtained in each factor and in the global result was calculated, with the lowest possible score 1 and the highest 5. For data presentation, they were also dimensioned as suggested in a previous study as dissatisfied those whose score is below 2.5 and satisfied those whose score is above 2.5^(2)^.

The SADL research instrument consists of 15 questions, aiming to quantify the overall satisfaction with the use of CI. The aspects assessed by it are: Positive effects (6 questions, eg CI help to understand people talk?, CI was the best treatment option?), service and costs (3 questions), negative factors (3 questions, eg feeling when you can't get the desired volume) and personal image (3 questions), by characterizing the overall score and the four subscale scores. Each question has 7 possible answers ranging from “not at all” to “extremely”, with the lowest possible score 1 and the highest 7. For analysis, the sum of the constituent questions of each scale was performed to obtain their mean, and for the overall value, the average scores obtained in each of the 4 scales of the inventory were summed. Considering scores below 3.5 as dissatisfied and above 3.5 as satisfied^(2)^.

WHOQOL-bref has twenty-six questions to which the answer is a 5-point likert scale. Is subdivided into domains and facets, where questions 1 and 2 are classified as facets and the other questions in domains. Domain I corresponds to the physical aspects (seven questions), domain II represents the psychological aspects (six questions), domain III elucidates questions of social relations (three questions), and domain IV specifies the perceptions of the environment (eight questions). The arithmetic mean of each domain and facets was calculated, the results were classified, as specified in the inventory manual, as follows: need to improve (score from 1 to 2.9); regular (from 3 to 3.9); good (4 to 4.9) and very good (score 5)^(16)^.

The application and completion of the pencil-and-paper inventories were performed in a room with the presence of a researcher. It has been given to each participant an explanation of the purpose of the inventories and any doubts on how to fill them up, being given the time necessary to fill them.

The allocation of individuals in the pre and postlingual groups was carried out as follows: when a hearing loss occurs before 3 years of age, consider a prelingual hearing loss. After that age, a postlingual hearing loss is considered^(17)^.

### Statistical analysis

All the variables were subjected to the normality tests of Shapiro-Wilk and Kolmogorov-Smirnovwhich. In order to statistically describe and compare the pre and postlingual groups, parametric tests were used when the distribution was Gaussian (Student's “T” test) and nonparametric tests (Mann Whitney) when the distribution was not Gausian. Spearman's nonparametric test was chosen for the correlation. Spearman's *ρ* shows that results close to -1 have a negative correlation, close to 0 have no correlation and close to 1 have a positive correlation. The level of significance of this analysis was 5% or 0.05 for both normality and correlation tests

The MS-Excel spreadsheet, in its version of MS-Office 2013, was used to organize the data, and the GraphPadPrism statistical package, in its version 6.0, to obtain the results. The study participants were grouped into satisfied and dissatisfied for a better general understanding of the results except for the WHOQOL-bref assessment, where the percentage of answers obtained in each domain was rated.

## RESULTS

### Sample characterization

The sample consisted of 24 individuals, 75% female and 25% male, at the average age of 42.83 years and SD of 15.59. Regarding the moment of onset of deafness, 15 (62.5%) had postlingual hearing loss and 09 individuals (37.5%) had prelingual hearing loss. The other characteristics of the individuals are presented in Table 1.

**Table 1:** Characteristics of the individuals included in the study

	Total		Prelingual		Postlingual	
	Mean Range (SD)	n(%)	Mean Range(SD)	n(%)	Mean Range(SD)	n(%)
Age at onset of hearing loss (years)	16.8		0.36		26.67	
	0 - 66 (20.89)		0 - 3 (0.70)		6- 66 (20.94)	
CI at the age of (years)	38.67		28.33		44.87	
	13 - 72 (17.32)		16 - 43 (7.45)		13 - 72 (18.74)	
Time of hearing loss before CI (years)	21.72		28.11		17.89	
	10-53 (16.00)		14-43 (7.88)		10-53 (4.74)	
CI experience time (years)	4.33		5.17		4.8	
	1-13 (3.47)		1-10 (3.31)		1-13 (3.26)	
Etiology						
Unknown		17 (70.83%)		6 (66.67%)		11 (73.33%)
Genetic		1 (4.17%)		0		1 (6.67%)
Meningitis		6 (25.00%)		3 (33.33%)		3 (20.00%)
Implant Type						
Cochlear		17 (70.80%)		5 (55.56%)		12(79.99%)
AdvancedBionics		2 (8.30%)		1 (11.11%)		1 (6.67%)
Medel		4 (16.70%)		3 (33.33%)		1 (6.67%)
Oticon Medical		1 (4.20%)		0		1 (6.67%)
Hours of daily use						
Less than 8 hours		2 (8.33%)		0		2 (13.33%)
More than 8 hours		22 (91.67%)		9 (100%)		13 (86.67%)
Currently under speech therapy*		22 (91.66%)		8 (88.88%)		14 (93.33%)

* Two individuals did not undergo speech therapy after CI, one from the prelingual group and one from the postlingual group

Regarding the distribution between the sexes, it is observed that in the postlingual group the concentration of women is higher than that observed in the prelingual group, thirteen (86.7%) in the postlingual group and five (55.6%) in the prelingual group.

Patients in the postlingual group were significantly older than the prelingual group (p = 0.001), with a mean age of 48.07 (range: 19 to 76) and SD of 17.21 compared with 34.11 (range: 26 to 45) and SD of 6.45 in the prelingual group.

The age at which patients had had their cochlear implantation was significantly higher in the postlingual group (p = 0.020). The time of sensory deprivation was longer in the prelingual group, however, this difference was not statistically significant (p = 0.075). CI experience time was similar between the two groups (p = 0.26).

### Satisfaction with cochlear implants

Regarding the SADL and IOI-CI surveys the median score, as well as the minimum and maximum values found in the total sample, prelingual group and postlingual group and the significance value of the comparison between groups are described in Table 2.

**Table 2:** Description of overall score. subscales. and SADL and IOI – CI factors of study participants

	Total	Prelingual	Postlingual	P value
Dimensions SADL	Median	Median	Median	
	(Total range)	(Total range)	(Total range)	
Global	5.30	5.12	5.54	0.65
	(1.20-6.60)	(3.58-6.42)	(2.33-6.50)	
Positive Effects	5.75	5.67	5.83	0.38
	(2.30-7.00)	(3.00-7.00)	(2.33-7.00)	
Negative Factors	5.00	4.33	5.00	0.53
	(1.30-6.70)	(2.33-6.33)	(1.33-6.67)	
Personal Image	5.85	5.33	6.67	0.07
	(3.30-7.00)	(3.33-7.00)	(3.67-7.00)	
Services and costs	4.85	5.33	4.67	0.53
	(1.30-7.00)	(2.67-7.00)	(1.33-7.00)	
Dimensions IOI- CI	Median	Median	Median	P value
	(Total range)	(Total range)	(Total range)	
Global	4.30	4.25	4.21	0.87
	(2.60-5.00)	(3.37-4.54)	(2.46-5.00)	
Factor 1	4.80	4.75	4.75	0.93
	(3.00-5.00)	(3.75-5.00)	(3.00-5.00)	
Factor 2	3.70	3.67 (1.67-5.00)	3.67	0.65
	(1.70-5.00)		(1.67-5.00)	

In the SADL inventory, the highest satisfaction scores were found in the subscales referring to positive and personal image factors. The lowest satisfaction scores were found in the subscales referring to the negative and the service and cost factors.

In the IOI-CI inventory, the highest satisfaction score was found in Factor 1 for the relationship between the user and their cochlear implant and the lowest satisfaction score was found in Factor 2 for the user 's relationship with their social environment.

The most frequently reported negative factors were the cost-effective and competitive noise, observed in the negative factors and the services and costs of the SADL inventory.

When compared to the scores of each of the surveys, the pre and postlingual groups did not present significant difference regarding cochlear implant satisfaction in all dimensions and factors of the SADL and IOI-CI inventory.

The analysis of the degree of global satisfaction with the CI through the IOI-CI demonstrated that 100% of the users were satisfied with the device. Analyzing the SADL, 87.5% of the participants demonstrated to be satisfied with the device and 12.5% dissatisfied, with the degree of satisfaction in the prelingual group of 100% and in the postlingual group of 80%.

No statistically significant difference was found between the proportion of satisfied and dissatisfied patients in the pre and postlingual group in IOI-CI, and a higher proportion of satisfied patients in the prelingual group in SADL was found.

### Quality of life

The results of the total sample regarding the quality of life analysis by the WHOQOL-bref assessment are presented in Table 3.

**Table 3:** Scores of each domain of the WHOQOL-bref assessment of the study participants

	Total	Prelingual	Postlingual	
Domain	Median	Median	Median	P value
	(Total range)	(Total range)	(Total range)	
I-Physical	4.00	3.86	4.00	0.82
	(2.14-5.14)	(3.14-4.86)	(2.42-4.71)	
II-Psychological	3.92	3.83	4.00	0.68
	(2.50-4.67)	(3.33-4.33)	(2.83-4.83)	
III-Social relations	4.00	4.00	3.67	0.65
	(2.00-5.00)	(3.00-4.66)	(2.00-5.00)	
IV-Environment	3.57	4.00	3.50	0.27
	(2.00-4.38)	(2.25-4.37)	(2.00-4.62)	
V- Facets	4.00	4.04	4.00	0.28
	(2.00-5.00)	(3.23-4.69)	(2.00-4.62)	

When comparing each constituent domain of the WHOQOL-bref assessment the pre and postlingual groups did not present significant differences.

**Figure 1:**
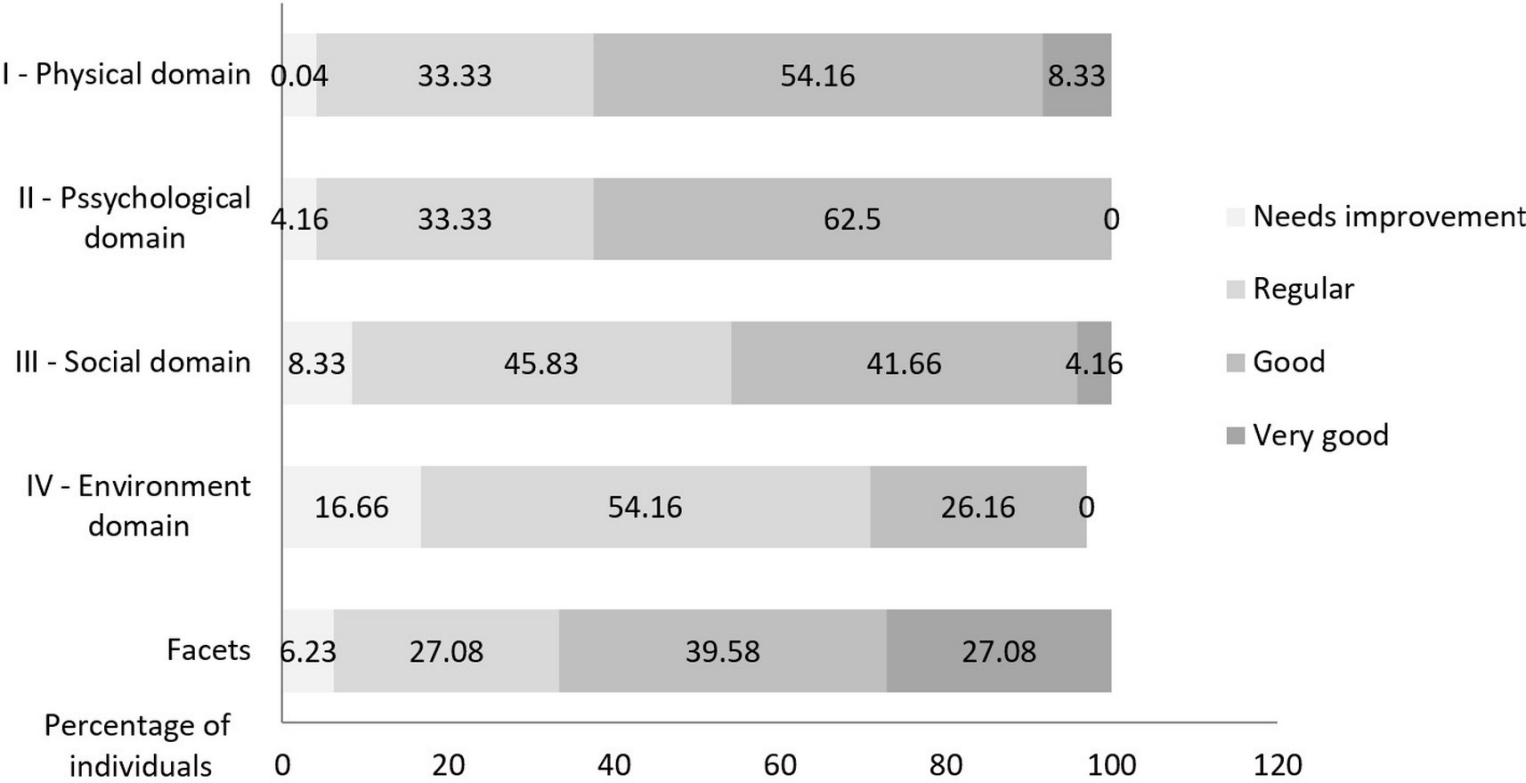
Percentages of responses to the WHOQOL-bref assessment

In the WHOQOL- bref assessment the results show us that users are satisfied with their quality of life. For a better understanding of the results, we present in Figure 1 the percentage of answers divided into “needs improvement”, “regular”, “good” and “very good”.

The highest scores were obtained in domain I, which refers to the physical domain, and in domain II, which refers to the psychological domain. We had the lowest scores in domain IV regarding the environment.

### Correlation between characteristics of the individuals and satisfaction with ci and quality of life

The analysis of the total sample, of the prelingual and postlingual groups showed that the older the patient (p=0.961; p=0.869; p=0.994) and the later the hearing loss (p=0.646; p=0.585; p=0.531) and the CI occurred (p=1.000; p=1.000; p=1.000), the greater the degree of satisfaction with the device observed through the SADL questionnaire. This correlation was not observed when analyzing the results of the IOI-CI.

In the prelingual group, it was possible to correlate the time of hearing loss before CI with the degree of satisfaction with the CI through the SADL, which is a positive correlation (p=1.000). In the postlingual group, it was observed that the individuals who did post implant speech therapy had greater satisfaction with their device, observed in the IOI-CI (p=0.537).

The activation time of the CI was negatively correlated with the degree of satisfaction with the device measured through the SADL (p=-0.617), analyzing the total sample.

Although there was greater satisfaction with the device in specific groups in the sample, there was no better quality of life among these individuals compared to the others.

## DISCUSSION

Assessment of user satisfaction is provided for in the Ordinance of the General Guidelines for specialized care for people with hearing impairment in the Unified Health System (SUS), as part of the monitoring of CI patients. Knowing the positive and negative points regarding the use of CI is of paramount importance for enabling and rehabilitating individuals with HL^(2)^.

The need for specific studies on quality of life in this population has recently been highlighted, as traditional speech recognition tests do not reflect this aspect^(1,16)^. There have been efforts to develop different surveys in the seeking of quality of life in this specific population^(11)^.

Studies already published show the effectiveness of CI as a treatment for individuals with severe to profound hearing loss, and that the degree of satisfaction stands out in relation to the dissatisfaction presented by CI users^(2,18)^.

Comparison between groups showed that even individuals with prelingual hearing loss had a high degree of satisfaction with CI. Previous studies agree that if selected according to the recommendations for selecting candidates for cochlear implant, patients with prelingual hearing loss benefit from the use of CI^10^. This fact deserves highlight in the present study because the individuals had a long duration of sensory deprivation. It is worth noting that the postoperative patients who underwent speech therapy had a higher degree of satisfaction with the device.

Knowledge of the negative factors presented by CI users, as analyzed in the SADL, is perhaps one of the most relevant returns in the satisfaction survey. Considering that users' complaints, whether psychological or auditory, can influence and be a determining factor for treatment abandonment^(2,19)^.

A significant number of dissatisfaction related to CI services and costs in general is observed^(2)^. Several studies have shown that at the public health level, CI is a cost-effective alternative in both high-income countries^(20)^ and developing countries^(21,22)^. Factors such as unilateral or bilateral implantation and candidates receiving the device still remain a controversial subject^(23)^.

Despite the possibility that CI can provide in reaching high levels of understanding, research reveals that CI users are still experiencing considerable difficulty in noisy environments^(24)^. In IOI-CI the lowest satisfaction score was found in Factor 2 of the inventory, which refers to the user's relationship with their social environment. From the reports of users during data collection, it was observed that this result was influenced by the degree of difficulty they still encounter in competitive noise situations using cochlear implant.

Hearing difficulty in noisy environments is associated with the ability of the auditory pathway to encode temporal envelope modulations due to the effect of degenerative processes associated with hearing loss^(25)^. Post-Hearing auditory training becomes essential; however, only a few studies address the perception of speech in a noisy environment as part of this training. Regarding this complaint, significant improvement has been demonstrated after five sessions of specific training^(26)^.

With the use of the device, adult users report considerable improvement in their hearing performance and communication difficulties, which allows the restoration of professional and social activities^(9)^.

The last question of IOI-CI questionnaire asked, considering everything, how they thought their cochlear implant changed their joy of living or rejoice in life, the vast majority of respondents claimed that CI provided them with much more joy of living. These results are also proven by other authors^(3)^. Self-esteem, social relationships and independence are negatively affected in the lives of people with hearing loss. CI for these people can improve these factors and favor their personal, social, family relationships and autonomy^(27,28)^.

The use of the CI electronic device reflects directly on the individual's quality of life and may benefit him/her in social and family life aspects. A previous study has shown that even individuals well adapted to hearing aids demonstrate improved quality after performing the cochlear implant^(29)^.

Cochlear implant users demonstrated good quality of life, and it was even demonstrated in a study with the WHOQOL-bref that the CI user participants had scores very close to the maximum score that represents a satisfactory quality of life resembling the results found in the participants without hearing loss^(11)^. It is important to point out that when comparing the quality of life of CI users with healthy adults, this group has significantly lower scores, demonstrating that the effects of hearing loss affect the quality of life even in implanted patients^(27)^.

Traditionally, patients who use CI are evaluated using tests of speech recognition skills. These measures are poorly correlated with improvement in communication, social, emotional aspects and quality of life^(11)^. Thus, studies have concentrated efforts on verifying the demographic and auditory factors that influence the individual's satisfaction with the cochlear implant, these factors still remain controversial^(14)^.

The individual's age was related to greater satisfaction with the device in both the prelingual and postlingual groups. This data differs from that previously reported^(14)^.

Among the hearing factors, longer duration of hearing loss before CI was associated with greater satisfaction with the device in prelingual group. Perhaps this fact can be justified by the satisfaction generated by reentering the world of hearing after a long time. This greater satisfaction with the device, however, did not reflect a significant improvement in quality of life. As it differs from a previous study that reported the relationship between these data to be unexpected^(14)^.

When comparing the results of the three surveys applied in this research, the pre and postlingual groups showed no difference in relation to the achieved scores. This fact reiterates the option to perform cochlear implant surgery in prelingual patients even with a long time of sensory deprivation, provided that they meet the selection criteria for the use of a cochlear implant that includes speech therapy by the verbal auditory therapy and appropriate motivation for cochlear implantation and for the rehabilitation process^(30)^.

## CONCLUSIONS

The participants had a high level of satisfaction with the use of cochlear implants. The longer the sensory deprivation time, the greater the degree of satisfaction with the device.

The highest degree of satisfaction was reported with regard to personal image, positive effects, and how the users feel about their CI. The lowest degree of satisfaction was reported regarding the cost-benefit of the CI and the competitive noise.

Sociodemographic and auditory factors interfere in the quality of life of these individuals and must be considered in their evaluation.

The use of the CI electronic device reflects directly on the individual's quality of life. Pre and postlingual groups showed no difference in relation to the achieved scores.
